# Outcome Prediction in Patients with Severe COVID-19 Requiring Extracorporeal Membrane Oxygenation—A Retrospective International Multicenter Study

**DOI:** 10.3390/membranes11030170

**Published:** 2021-02-27

**Authors:** Alexander Supady, Jeff DellaVolpe, Fabio Silvio Taccone, Dominik Scharpf, Matthias Ulmer, Philipp M. Lepper, Maximilian Halbe, Stephan Ziegeler, Alexander Vogt, Raj Ramanan, David Boldt, Stephanie-Susanne Stecher, Andrea Montisci, Tobias Spangenberg, Olivier Marggraf, Chandra Kunavarapu, Lorenzo Peluso, Sebastian Muenz, Monica Buerle, Naveen G. Nagaraj, Sebastian Nuding, Catalin Toma, Vadim Gudzenko, Hans Joachim Stemmler, Federico Pappalardo, Georg Trummer, Christoph Benk, Guido Michels, Daniel Duerschmied, Constantin von zur Muehlen, Christoph Bode, Klaus Kaier, Daniel Brodie, Tobias Wengenmayer, Dawid L. Staudacher

**Affiliations:** 1 Department of Medicine III (Interdisciplinary Medical Intensive Care), Medical Center, Faculty of Medicine, University of Freiburg, Hugstetter Strasse 55, 79106 Freiburg, Germany; daniel.duerschmied@universitaets-herzzentrum.de (D.D.); constantin.vonzurmuehlen@universitaets-herzzentrum.de (C.v.z.M.); christoph.bode@universitaets-herzzentrum.de (C.B.); tobias.wengenmayer@universitaets-herzzentrum.de (T.W.); dawid.staudacher@universitaets-herzzentrum.de (D.L.S.); 2 Department of Cardiology and Angiology I, Heart Center, University of Freiburg, 79106 Freiburg, Germany; klaus.kaier@uniklinik-freiburg.de; 3 Heidelberg Institute of Global Health, University of Heidelberg, 69120 Heidelberg, Germany; 4 Methodist Hospital, San Antonio, TX 78229, USA; jeff.dellavolpe@gmail.com (J.D.); C.kunavarapu@gmail.com (C.K.); 5 Department of Intensive Care, Erasme Hospital, Université Libre de Bruxelles, 1070 Brussels, Belgium; ftaccone@ulb.ac.be (F.S.T.); lorenzopeluso80@gmail.com (L.P.); 6 SLK-Hospital Heilbronn, 74078 Heilbronn, Germany; Dominik.Scharpf@slk-kliniken.de (D.S.); Sebastian.Muenz@slk-kliniken.de (S.M.); 7 RKH Hospital Ludwigsburg, 71640 Ludwigsburg, Germany; Matthias.Ulmer@rkh-kliniken.de (M.U.); monica.buerle@rkh-kliniken.de (M.B.); 8 Department of Internal Medicine V—Pneumology, Allergology and Critical Care Medicine, Saarland University Medical Center and University of Saarland, D-66421 Homburg, Germany; philipp.lepper@uks.eu; 9 Heart Center, University Hospital Zurich, 8006 Zurich, Switzerland; Maximilian.Halbe@usz.ch (M.H.); NaveenGaddehosur.Nagaraj@usz.ch (N.G.N.); 10 Department of Anesthesiology, Intensive Care Medicine and Pain Management, Hospital Ibbenbueren, 49477 Ibbenbueren, Germany; s.ziegeler@klinikum-ibbenbueren.de; 11 Department of Medicine III, University Clinic Halle (Saale), 06097 Halle (Saale), Germany; alexander.vogt@uk-halle.de (A.V.); sebastian.nuding@uk-halle.de (S.N.); 12 University of Pittsburgh Medical Center (UPMC), Pittsburgh, PA 15213, USA; Ramananrr@upmc.edu (R.R.); tomacx@UPMC.EDU (C.T.); 13 UCLA Healthcare System, Los Angeles, CA 90095, USA; DBoldt@mednet.ucla.edu (D.B.); VGudzenko@mednet.ucla.edu (V.G.); 14 Medical Department II, LMU Hospital Munich, 80331 Munich, Germany; StephanieSusanne.Stecher@med.uni-muenchen.de; 15 Istituto Clinico Sant’Ambrogio, University of Milan, 20149 Milan, Italy; montisci.andrea@yahoo.it; 16 Department of Cardiology, Angiology and Intensive Care, Marien Hospital Hamburg, 22087 Hamburg, Germany; t.spangenberg@asklepios.com; 17 Asklepios Clinic North, 22417 Hamburg, Germany; o.marggraf@asklepios.com; 18 Medical Department III, LMU Hospital Munich, 80331 Munich, Germany; joachim.stemmler@med.uni-muenchen.de; 19 Department of Anesthesia and Intensive Care, IRCCS ISMETT, UPMC Italy, 90127 Palermo, Italy; fedepappa.71@gmail.com; 20 Department of Cardiovascular Surgery, Heart Center, University of Freiburg, 79106 Freiburg, Germany; georg.trummer@universitaets-herzzentrum.de (G.T.); christoph.benk@universitaets-herzzentrum.de (C.B.); 21 Department of Acute and Emergency Care, St. Antonius Hospital Eschweiler, 52249 Eschweiler, Germany; Guido.Michels@SAH-ESCHWEILER.DE; 22 Institute of Medical Biometry and Statistics, Faculty of Medicine, University of Freiburg, 79104 Freiburg, Germany; 23 Department of Medicine, Columbia University College of Physicians and Surgeons/New York-Presbyterian Hospital, New York, NY 10032, USA; hdb5@cumc.columbia.edu

**Keywords:** acute respiratory distress syndrome, extracorporeal membrane oxygenation, COVID-19, SARS-CoV-2

## Abstract

The role of veno-venous extracorporeal membrane oxygenation therapy (V-V ECMO) in severe COVID-19 acute respiratory distress syndrome (ARDS) is still under debate and conclusive data from large cohorts are scarce. Furthermore, criteria for the selection of patients that benefit most from this highly invasive and resource-demanding therapy are yet to be defined. In this study, we assess survival in an international multicenter cohort of COVID-19 patients treated with V-V ECMO and evaluate the performance of several clinical scores to predict 30-day survival. Methods: This is an investigator-initiated retrospective non-interventional international multicenter registry study (NCT04405973, first registered 28 May 2020). In 127 patients treated with V-V ECMO at 15 centers in Germany, Switzerland, Italy, Belgium, and the United States, we calculated the Sequential Organ Failure Assessment (SOFA) Score, Simplified Acute Physiology Score II (SAPS II), Acute Physiology And Chronic Health Evaluation II (APACHE II) Score, Respiratory Extracorporeal Membrane Oxygenation Survival Prediction (RESP) Score, Predicting Death for Severe ARDS on V-V ECMO (PRESERVE) Score, and 30-day survival. Results: In our study cohort which enrolled 127 patients, overall 30-day survival was 54%. Median SOFA, SAPS II, APACHE II, RESP, and PRESERVE were 9, 36, 17, 1, and 4, respectively. The prognostic accuracy for all these scores (area under the receiver operating characteristic—AUROC) ranged between 0.548 and 0.605. Conclusions: The use of scores for the prediction of mortality cannot be recommended for treatment decisions in severe COVID-19 ARDS undergoing V-V ECMO; nevertheless, scoring results below or above a specific cut-off value may be considered as an additional tool in the evaluation of prognosis. Survival rates in this cohort of COVID-19 patients treated with V-V ECMO were slightly lower than those reported in non-COVID-19 ARDS patients treated with V-V ECMO.

## 1. Introduction

The severe acute respiratory syndrome coronavirus 2 (SARS-CoV-2) may cause severe pneumonia and life-threatening acute respiratory distress syndrome (ARDS) [1]. Hospitalized coronavirus disease 2019 (COVID-19) patients require invasive mechanical ventilation in about 17% of cases and veno-venous extracorporeal membrane oxygenation therapy (V-V ECMO) is applied in 1% [2,3]. 

Even though the SARS-CoV-2 pandemic hit first many well-developed countries, intensive care mortality was reported to be high (between 26% and 61%) [1,2,4,5]. Survival of COVID-19 patients with severe respiratory failure treated with V-V ECMO ranges around 60%, according to recent studies [6,7]. 

Recommendations for the use of V-V ECMO in COVID-19-related ARDS are being developed, though under continuous review [8,9,10]. So far, little data exist to guide clinicians in the decision algorithm of patients eligible for V-V ECMO in COVID-19. The unanswered key questions are patient selection and timing of V-V ECMO initiation [11,12]. Due to the novelty of the disease, it is unclear whether scores such as the Sequential Organ Failure Assessment (SOFA) Score, the Simplified Acute Physiology Score II (SAPS II), Acute Physiology And Chronic Health Evaluation II (APACHE II) Score, Respiratory Extracorporeal Membrane Oxygenation Survival Prediction (RESP) Score, and the Predicting Death for Severe ARDS on V-V ECMO (PRESERVE) Score are suitable to guide treatment decisions in patients with COVID-19-related ARDS requiring V-V ECMO support [13,14,15,16,17,18].

The aim of the present study is to describe relevant baseline and treatment characteristics and the outcomes in a large international cohort of COVID-19-related ARDS patients who received V-V ECMO therapy. Second, we test the performance of different clinical scores (SOFA, SAPS II, APACHE II, RESP, and PRESERVE) to predict 30-day survival in this patient cohort. 

## 2. Methods

### 2.1. Study Design

The study is an investigator-initiated retrospective non-interventional international multicenter registry study. The study was registered with ClinicalTrials.gov (NCT04405973). All data were collected retrospectively from patient records at the participating centers. Data from 15 centers in the United States, Germany, Belgium, Switzerland, and Italy were collected (Table 1). 

At the participating centers, all patients with reverse transcriptase polymerase chain reaction (rtPCR)-confirmed SARS-CoV-2 infection, who received V-V ECMO, were included. ECMO implantations were performed from 12 March 2020, through 5 June 2020, i.e., during the first wave of the pandemic. Data collection was performed from 19 May 2020, through 6 July 2020. As data were collected retrospectively, no interventions were applied for the purpose of this study and all patients included were treated per protocol as part of local standard care. The study conforms to the ethical guidelines of the 1975 Declaration of Helsinki and was approved by the leading institutional ethics committee of the University of Freiburg (EK 329/20). Due to the retrospective and observational nature of the study and anonymous data evaluation, the need for informed consent was waived. The RECORD statement was followed for the reporting of this study [19].

### 2.2. Data Collection and Management

In each participating center, patient files and hospital records were screened for patients with SARS-CoV-2 infection, severe respiratory failure, and V-V ECMO treatment. Gender, age, initiation, and decannulation from ECMO and 30-day survival were recorded. Furthermore, medical history, laboratory, and treatment parameters for calculation of SOFA, SAPS II, APACHE II, RESP, and PRESERVE scores before initiation of ECMO were compiled [13,14,15,16,17,18]. All data were derived directly from the participating hospitals’ patient files and entered into an electronic chart (Microsoft Excel 2010, Microsoft Corp., Redmond, WA, USA) by members of the study team. 

**Figure 1:**
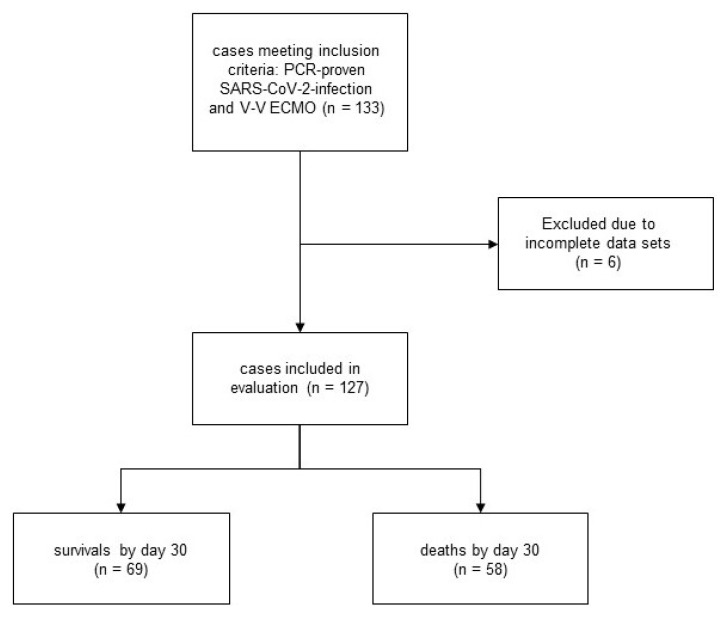
CONSORT flow-chart illustrating patient recruitment, case exclusion, and number of survivors and non-survivors in the study collective.

Data were cross-checked after entry by a second study team member for accuracy. Patients were included for data evaluation if all data required for this study were available (Figure 1). For estimation of inspired oxygen fraction in patients not mechanically ventilated, we used the approach described in previous studies to determine the concentration of oxygen supply [14,20,21]. In case of singular missing non-endpoint data, median imputation was used. This was necessary for a total of 0.9% of values. Six patients had to be excluded from evaluation due to missing scores and endpoint data.

### 2.3. Definitions

Thirty-day survival was defined as survival until at least the beginning of the 30th day after implantation of V-V ECMO. Age was defined as age at the time of V-V ECMO implantation. Scores were calculated from clinical data from the last 24 h before V-V ECMO implantation. Data of preexisting medical conditions used for calculation of the scores may or may not have been available to clinicians when the patients were treated. 

### 2.4. Statistical Methods

Statistical analyses were performed using GraphPad Prism 8 (GraphPad Software, San Diego, CA, USA). Follow-up on 30-day survival was complete for all patients and Kaplan–Meier plots were used to visualize 30-day survival, and significance between two groups was calculated with the Log-rank (Mantel-Cox) test. The impact of continuous and categorical baseline characteristics on 30-day mortality was evaluated using Mann-Whitney tests for continuous variables and Chi-square tests for categorical variables. Comparison of survival rates between different score quartiles was performed using one-way ANOVA, followed by Tukey’s multiple comparisons test. In all evaluations, a *p*-value at or below 0.05 was considered statistically significant. Discrimination abilities of scores were assessed with Receiver Operating Characteristic (ROC) curves. The Area Under the Curve (AUC) was calculated and the maximum Youden’s Index (J = sensitivity + specificity − 1) was employed to define an optimal cut-off point.

## 3. Results

### 3.1. Patients

A total of 133 patients met the inclusion criteria; 6 patients had to be excluded due to missing data and 127 patients with confirmed SARS-CoV-2 infection and V-V ECMO were included in the final analysis (Figure 1). 

**Figure 2:**
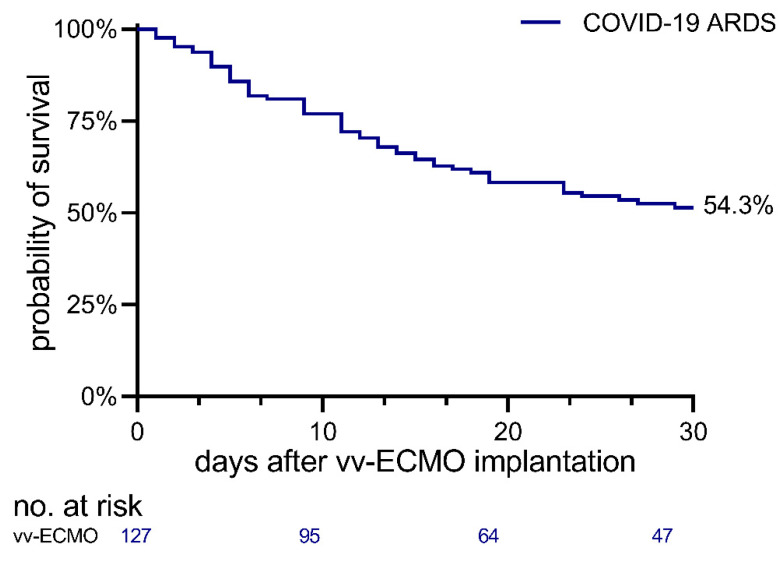
Kaplan–Meier curve displaying the survival over the course of the 30-day study period in the entire cohort.

The median age was 59 years, 21.3% of the patients (27/127) were women. The median body mass index (BMI) was 29 kg/m^2^; 54.3% of all patients (69/127) survived until day 30 after ECMO implantation, 45.7% (58/127) survived until day 60. Patients who survived (day 30) were younger (58 vs. 61 years, *p* = 0.016, Figure 2, Table 2). 

### 3.2. Ventilator Settings Blood Gas Analysis before ECMO

Patients in both groups, survivors and non-survivors, had similar ventilator settings prior to ECMO implantation. Furthermore, partial arterial pressure of oxygen and carbon dioxide and pH were comparable (Table 2). 

### 3.3. Pre-Existing Conditions

No significant differences in pre-existing conditions have been found for the two groups. Considering only severe pre-conditions like, e.g., heart failure NYHA IV or biopsy-confirmed liver cirrhosis, only a few patients in each group, survivors and non-survivors, respectively, suffered from any of these pre-conditions (Table 2). 

### 3.4. Intensive Care and ARDS Treatment

The frequency of prone positioning (71.0 vs. 77.6%, *p* = 0.404) and the frequency of neuromuscular blockage before ECMO implantation (49 vs. 57%, 0.392) were high in both groups. Prone positioning, and median PaO_2_/FiO_2_ ratio (70.2 vs. 72.5 mmHg, *p* = 0.984) did not differ between survivors and non-survivors. The median duration of invasive mechanical ventilation was shorter in survivors (3 vs. 6 days, *p* = 0.006). The use of nitric oxide and bicarbonate was equally rare in both groups (Table 2).

### 3.5. Clinical Scores 

**Figure 3:**
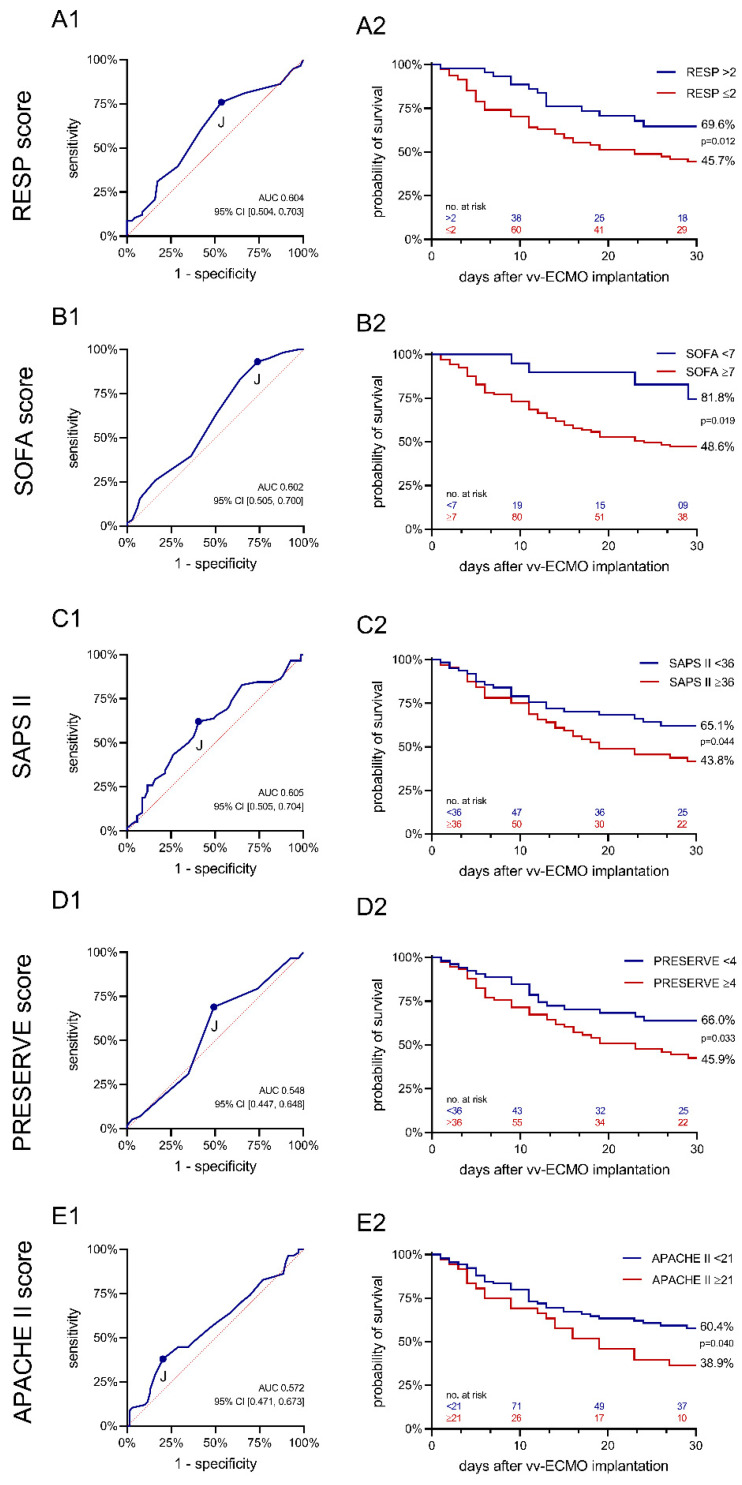
Receiver operating characteristic (ROC) analyses for the scores analyzed and Kaplan-Meier curves displaying the survival in the groups above and below the cut-off values, respectively, over the course of the 30-day study period. A1: ROC analysis RESP score. A2: Kaplan-Meier curve RESP score. B1: ROC analysis SOFA score. B2: Kaplan-Meier curve SOFA score. C1: ROC analysis SAPS II. C2: Kaplan-Meier curve SAPS II. D1: ROC analysis PRESERVE score. D2: Kaplan-Meier curve PRESERVE score. E1: ROC analysis APACHE II score. E2: Kaplan-Meier curve APACHE II score.

Median score results for SOFA, RESP, PRESERVE, SAPS II, and APACHE II are displayed in Table 2. Only the SOFA score and the SAPS II were significantly different between survivors and non-survivors. In a receiver operating characteristic (ROC) analysis for prediction of 30-day mortality, the area under ROC for SOFA was 0.602, for RESP 0.603, for PRESERVE 0.548, for SAPS II 0.605, and for APACHE II 0.572, respectively (Figure 3). 

The optimum cut-off point for predicting mortality was 2 for the RESP score. Drawing Kaplan–Meier graphs for cases on both sides of this cut-off (RESP > 2 and RESP ≤ 2, respectively) revealed significantly different survival rates for both groups (survival_RESP>2_ = 69.6%, survival_RESP ≤ 2_ = 45.7%, *p* = 0.010). 

The optimum cut-off point for predicting mortality was 7 for the SOFA score. Drawing Kaplan–Meier graphs for cases on both sides of this cut-off (SOFA ≥ 7 and SOFA < 7, respectively) revealed significantly different survival rates for both groups (survival_SOFA ≥ 7_ = 48.6%, survival_SOFA < 7_ = 81.8%, *p* = 0.010). 

The cut-off points and survival rates for all other scores are given in Figure 3. All comparisons of survival rates above and below the cut-off points revealed significant differences. 

The direct comparison confirms the weak relationship between score levels and observed survival (see Supplementary Material). Analysis of score quartiles did not show statistically significant differences in observed 30-day survival according to score quartile. The only significantly different survival rates were found in the lower quartiles of the PRESERVE score, all other survival rates were similar. 

## 4. Discussion

We report the results from an international multicenter registry of COVID-19-related ARDS patients treated with V-V ECMO. In our cohort, we observed a 30-day survival rate of 54% and a 60-day survival of 46%. The second major finding of our study is that well-established intensive care unit (ICU) scores (SOFA, SAPS II, APACHE II, RESP, and PRESERVE) are not useful to guide therapy decisions with regard to V-V ECMO in COVID-19-related ARDS. These observations, however, have to be considered preliminary and interpreted cautiously since we evaluated only retrospective data from a small cohort. In the absence of a control group, we cannot compare our findings with non-COVID-19 patients or patients treated without ECMO, either.

Survival rates in this critically ill patient cohort are slightly lower than survival observed in non-COVID ARDS treated with V-V ECMO and similar to results from another large cohort treated with V-V ECMO in COVID-19 [6,22]. In a cohort in Paris, the probability of death at day 28 was 18% [7]. As a major difference compared to this cohort, our population was considerably older (59 years vs. 49 years) and prone positioning was used less often (74% vs. 94%). To take note, in our cohort, median duration of invasive mechanical ventilation prior to ECMO was significantly shorter in survivors compared to non-survivors (3 days vs. 6 days). These differences may explain, at least in part, higher mortality in our cohort (46%); all other baseline characteristics have been relatively similar in both cohorts. However, it is not possible to use our data to explain the causal influence of these differences on survival. Another reason for the better outcome in the Paris cohort may be the expertise and high volume of patients treated with ECMO in this center during the COVID pandemic and before.

Early in the pandemic, the role of V-V ECMO for the treatment of COVID-19 was challenged, and the observed poor results were even attributed to the deleterious effects of undergoing ECMO [23,24]. Furthermore, concerns about resource limitations in light of overwhelmed health facilities and health systems have been raised [25,26].

Together with the results from previous observations of patient cohorts affected by severe COVID-19 ARDS and treated with V-V ECMO, our results may encourage further use of V-V ECMO in the treatment of severe COVID-19 ARDS and further research in this field.

Assuming that there is a role for V-V ECMO in the treatment of severe COVID-19-related ARDS, this raises questions of how best to select patients for this highly invasive treatment with the potential for serious complications. In the context of a pandemic, where available equipment and human resources may be limited, physicians will need guidance to assist them in making treatment decisions and help them select patients for resource- and staff-challenging treatment options like V-V ECMO. Our results, however, cannot support the use of scores like SOFA, SAPS II, APACHE II, RESP, or PRESERVE to solely guide this decision. According to ROC-analyses, all evaluated scores performed poorly in predicting mortality in COVID-19 ARDS patients. This is also true for the RESP score and the PRESERVE score that have been developed specifically for outcome prediction of patients requiring V-V ECMO.

For all scores, no differences in observed survival between low, medium, and high score values can be shown—only in the lower quartiles of the PRESERVE score, significantly different survival rates were found (Figure S1(A1–E1) and Table S1, see Supplementary Material). Nevertheless, we calculated the optimal cut-off point for each score. Survival of patients scoring above the respective cut-off value was between 60 and 80 percent, while patients scoring below this cut-off value showed a significantly reduced survival between 40 and 50 percent (see Figure 3). Whereas a direct comparison of the distribution of score levels between survivors and non-survivors also shows a distribution, which does not differ significantly between the groups, it can also be seen here that beyond the cut-off values determined, the number of survivors outweighs the number of deaths observed (Figure S1(A3–E3), see Supplementary Material).

We can only speculate on reasons for the poor performance of the scores in severe COVID-19-related ARDS for patients treated with V-V ECMO. Most of these scores have been developed and validated decades ago. Considering recent advances in critical care medicine, the outcome predictions derived from these historic validation cohorts ought to be viewed with caution. Furthermore, even though there is increasing evidence showing multiple organ affinity by SARS-CoV-2 infections (e.g., liver, heart, kidney, coagulation system), in our study, the affinity for the lungs was paramount [2]. In this context, SAPS II, APACHE II, and SOFA may underestimate the severity of the disease when it is focused on a single organ while others are still functioning well.

Since the RESP score and the PRESERVE score have been developed specifically for ARDS patients receiving V-V ECMO, the considerations above cannot account for their poor performance. The limited value of these scores for the prediction of mortality in our cohort may rather be due to specifics of COVID-19 ARDS and its pathophysiological differences compared to other known forms of pneumonia [27]. Respiratory failure in severe COVID-19 can be profound and treatment is complex—patients may require a long time on a mechanical ventilator, repeated prone positioning over several days and ventilation invasiveness is high [2]. This complexity is not reflected by the RESP score and the PRESERVE score.

Despite the poor performance of the scores in our cohort, we were able to define cut-off values that may play a role in the prognosis assessment of these patients. However, they cannot be used for triage and decision-making for and against ECMO therapy. This finding should be a basis for further research.

The results of this retrospective international multicenter registry study may help to better understand the role of V-V ECMO in the treatment of severe COVID-19 ARDS. The observed survival of more than 50% in this critically ill patient population is close to the survival observed in non-COVID ARDS treated with V-V ECMO and also to survival rates observed in critically ill COVID-19 patients treated without ECMO; a recent meta-analysis on V-V ECMO in non-COVID ARDS even described reduced mortality in V-V ECMO compared to conventional therapy [2,22,28]. Further research is needed to better understand the selection of suitable patients as the scores examined in our study have only limited value in the decision-making process.

### 4.1. Limitations

The study design is a single-arm retrospective multicenter registry; therefore, we cannot compare our results to a control group. Furthermore, the number of patients included is rather low, limiting the power of our results and generalizability. Since patients were included retrospectively, we cannot rule out selection bias. In addition to that, we only report 30- and 60-day-survival data, which is less meaningful and most likely higher than hospital survival or even longer observation periods.

As only COVID-19 patients treated with V-V ECMO were included in this registry, we cannot draw any inference on the potential outcome of these patients without V-V ECMO. Furthermore, we cannot directly compare the results from our observation with other studies or observations in non-COVID-19 ARDS treated with or without ECMO. The data presented here cannot be used for determining if a given patient should receive V-V ECMO.

In our cohort, survivors were treated shorter with invasive mechanical ventilation before ECMO than non-survivors. However, our data do not allow determining a time threshold for mechanical ventilation in COVID-19 after which initiation of ECMO is no longer reasonable. This should be further assessed in subsequent trials.

Potential differences in ECMO practices and criteria for ECMO at the different centers may limit the generalizability of our results. Moreover, different local treatment standards for ARDS, ECMO, and COVID-19 may have been applied in the participating centers (which may also have changed over time due to emerging new evidence). The trial was not designed to find out more generally about drivers of mortality or the effect of different treatments in this specific patient cohort. With respect to drug treatment, during the observation period, the results of the RECOVERY trial had not been published, therefore, dexamethasone had not been introduced as standard treatment. However, since we did not collect this information, we cannot report if patients have been treated with glucocorticoids which could have an impact on survival. Finally, our registry did not include treatment and outcome data after initiation of ECMO, besides survival. Therefore, we cannot analyze the response to ECMO after the initiation of ECMO.

### 4.2. Conclusions

1. Thirty-day-survival in COVID-19 patients treated with V-V ECMO and evaluated in our registry is 54 percent, encouraging further clinical research and application.
2. The use of scores for the prediction of mortality cannot be recommended for treatment decisions in severe COVID-19 ARDS and V-V ECMO should be considered if deemed beneficial as long as resources are available. Scoring results below or above a specific cut-off value may be considered as an additional tool in the evaluation of prognosis but cannot be used for triaging this patient population.

## Figures and Tables

**Table 1:** Participating centers. Cases included in the registry listed by treatment centers and countries.

Center	Country	Number of Cases
Methodist Hospital, San Antonio, Texas	United States	21
University Clinic Freiburg	Germany	15
Hôpital Erasme—ULB, Brussels	Belgium	15
Clinic Heilbronn	Germany	14
Clinic Ludwigsburg	Germany	11
Saarland University Medical Center	Germany	9
University Clinic Zurich	Switzerland	9
Clinic Ibbenbueren *	Germany	8
University Clinic Halle (Saale)	Germany	8
University of Pittsburgh Medical Center (UPMC)	United States	8
UCLA Healthcare System, Los Angeles	United States	5
LMU Hospital Munich	Germany	4
Istituto Clinico Sant’Ambrogio, University of Milan	Italy	3
Marien Hospital Hamburg	Germany	2
Asklepios Clinic North, Hamburg	Germany	1
Total number of cases		133

* Some patients have been transferred after V-V ECMO-implantation to a collaborating partner hospital (Karl-Hansen-Clinic in Bad Lippspringe, Germany) and follow-up data were collected there.

**Table 2:** Patient baseline (before initiation of extracorporeal membrane oxygenation therapy (ECMO)) characteristics as well as results for Sequential Organ Failure Assessment (SOFA), Simplified Acute Physiology Score II (SAPS II), Acute Physiology And Chronic Health Evaluation II (APACHE II), Respiratory Extracorporeal Membrane Oxygenation Survival Prediction (RESP), and Predicting Death for Severe ARDS on V-V ECMO (PRESERVE) scores in all patients and in survivors and non-survivors independently (median, IQR). The impact of continuous and categorical baseline characteristics on 30-day survival was evaluated using Chi-square and Mann–Whitney tests.

	Total	Non-Survivors (*n* = 58)	Survivors (*n* = 69)	*p*
**Patients**				
Number of patients, No. (%)	127 (100)	58 (46)	69 (54)	--
Female sex, No. (%)	27 (21)	10 (17)	17 (25)	0.314
Age [years], median (IQR)	59 (53–66)	61 (54–70)	58 (51–64)	0.019
BMI [kg/m^2^], median (IQR)	29 (26–35)	29 (26–35)	30 (25–34.4)	0.701
Evidence of SARS-CoV-2 (PCR), No. (%)	127 (100)	58 (100)	69 (100)	--
**Ventilation parameters and blood gas analysis before ECMO**, median (IQR)				
FiO_2_ [%]	100 (80–100)	100 (80–100)	96 (80–100)	0.929
PEEP [mbar]	14 (10–16)	15 (10–17)	13 (10–15)	0.077
Plateau pressure [mbar]	32 (26–35)	32 (28–35)	32 (24.5–34)	0.116
Driving pressure [mbar]	17 (13–21)	17 (13.75–21.25)	16 (12.5–20)	0.283
Breathing rate [1/min]	25 (21–30)	25.5 (20.5–32.5)	24 (20.5–30)	0.372
pH	7.3 (7.2–7.5)	7.3 (7.2–7.4)	7.3 (7.2–7.4)	0.980
pO_2_ [mmHg]	64 (52–76)	64 (53–78)	64 (52–76)	0.867
pCO_2_ [mmHg]	57 (45–67)	58 (45–70)	57 (44–64)	0.290
PaO_2_/FiO_2_ [mmHg]	70.2 (57.1–97.1)	72.05 (56.6–94.5)	70.2 (57.1–99.3)	0.984
**Pre-existing conditions**, No. (%)				
Heart failure NYHA IV	3 (2)	3 (5)	0 (0)	0.056
Chronic lung disease	13 (10)	9 (16)	4 (6)	0.072
Dialysis-dependent kidney failure	11 (9)	4 (7)	7 (10)	0.517
Hematologic malignancy	6 (5)	4 (7)	2 (3)	0.290
Solid malignant tumor	3 (2)	2 (3)	1 (1)	0.460
Metastatic cancer	1 (1)	0 (0)	1 (1)	0.357
Solid organ transplant	0 (0)	0 (0)	0 (0)	--
HIV	1 (1)	1 (2)	0 (0)	0.274
Liver cirrhosis	0 (0)	0 (0)	0 (0)	--
Immunosuppressive therapy	8 (6)	5 (9)	3 (4)	0.324
**Intensive care and ARDS treatment**, No. (%)				
Prone positioning before ECMO	94 (74)	45 (78)	49 (71)	0.404
Duration of invasive ventilation before ECMO [days], median (IQR)	5.0 (2.0–9.0)	6.0 (3.75–11.25)	3.0 (1.0–8.0)	0.006
Nitric oxide use before ECMO	6 (5)	4 (7)	2 (3)	0.290
Bicarbonate use before ECMO	11 (9)	7 (12)	4 (6)	0.211
Neuromuscular blockers before ECMO	67 (53)	33 (57)	34 (49)	0.392
On day 30 after ECMO initiation still on ECMO	24 (19)	0 (0)	24 (35)	--
On day 30 after ECMO initiation still on ICU	47 (37)	0 (0)	47 (68)	--
Renal replacement therapy before ECMO	24 (19)	11 (19)	13 (19)	0.986
Renal replacement therapy during ECMO	73 (57)	38 (66)	33 (48)	0.016
**Scores**, median (IQR)				
SOFA	9 (7–10)	9 (8–11)	9 (6–10)	0.045
RESP	1 (0–4)	1 (-1–2)	2 (0–4)	0.051
PRESERVE	4 (3–5)	4 (3–5)	3 (2–5)	0.389
SAPS II	36 (29–44)	38.5 (30–48)	34 (28–41)	0.042
APACHE II	17 (12–21)	17 (12–22)	16 (12–20)	0.164

## Data Availability

All data will be available from the corresponding author on reasonable request.

## Supplementary Materials

The following are available online at https://www.mdpi.com/2077-0375/11/3/170/s1. Figure S1: Rates of survival and number of cases for different score levels. Table S1: Comparison between rates of survival between four quartiles of the scores using one-way ANOVA and Tukey’s multiple comparisons test.
