# Supplementary material for: Outcome Prediction in Patients with Severe COVID-19 Requiring Extracorporeal Membrane Oxygenation—A Retrospective International Multicenter Study
Source: Membranes (Basel). 2021 Feb 27;11(3):170. doi: 10.3390/membranes11030170 (PMC7997249; doi:10.3390/membranes11030170)
Supplement: Supplementary file 1 [file membranes-11-00170-s001.pdf]

Supplementary material

Figure S1. Rates of survival and number of cases for different score levels. Rates of survival between four quartiles of SOFA, RESP, PRESERVE, SAPS II and APACHE II (A1-E1), number of cases in for the respective score levels in the entire cohort (A2-E2) and for survivors and deceased separately (A3-E3), ** p ≤0.01.

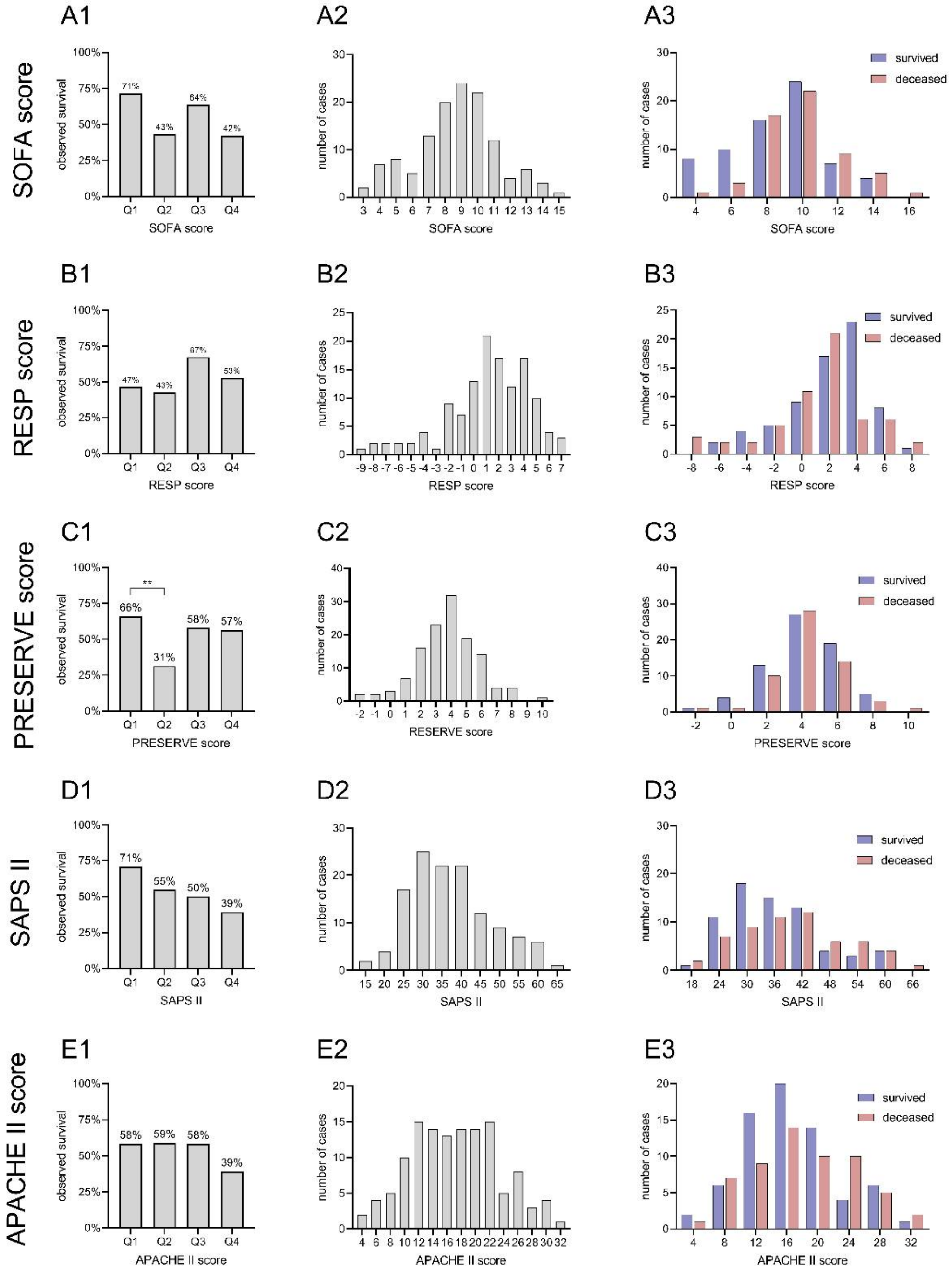

Table S1: Comparison between rates of survival between four quartiles of the scores using one-way ANOVA and Tukey's multiple comparisons test.

	One-way ANOVA		Tukey‘s multiple comparisons test	
	F	p	mean diff.	adjusted p
SOFA	2.982	0.034		
Q1 vs. Q2			28.25	0.0571
Q1 vs. Q3			7.792	0.9361
Q1 vs. Q4			29.12	0.1033
Q2 vs. Q3			-20.45	0.3808
Q2 vs. Q4			0.8741	0.9999
Q3 vs. Q4			21.33	0.4367
RESP	1.803	0.1502		
Q1 vs. Q2			3.654	0.9925
Q1 vs. Q3			-20.88	0.1985
Q1 vs. Q4			-6.430	0.9689
Q2 vs. Q3			-24.53	0.2418
Q2 vs. Q4			-10.08	0.9242
Q3 vs. Q4			14.45	0.7336
PRESERVE	3.481	0.0180		
Q1 vs. Q2			34.79	0.0094
Q1 vs. Q3			8.143	0.9233
Q1 vs. Q4			9.516	0.8615
Q2 vs. Q3			-26.64	0.2363
Q2 vs. Q4			-25.27	0.2324
Q3 vs. Q4			1.373	0.9997
SAPS II	2.182	0.0935		
Q1 vs. Q2			16.04	0.5450
Q1 vs. Q3			20.59	0.3308
Q1 vs. Q4			31.30	0.0670
Q2 vs. Q3			4.545	0.9824
Q2 vs. Q4			15.26	0.6254
Q3 vs. Q4			10.71	0.8355
APACHE II	1.087	0.3572		
Q1 vs. Q2			-0.6410	>0.9999
Q1 vs. Q3			0.000	>0.9999
Q1 vs. Q4			19.05	0.4328
Q2 vs. Q3			0.6410	>0.9999
Q2 vs. Q4			19.69	0.3875
Q3 vs. Q4			19.05	0.5201
